# Emergency department patients with weakness or fatigue: Can physicians predict their outcomes at the front door? A prospective observational study

**DOI:** 10.1371/journal.pone.0239902

**Published:** 2020-11-05

**Authors:** Stefan M. Herzog, Mirjam A. Jenny, Christian H. Nickel, Ricardo Nieves Ortega, Roland Bingisser

**Affiliations:** 1 Center for Adaptive Rationality, Max Planck Institute for Human Development, Berlin, Germany; 2 Science Communication Unit, Robert Koch Institute, Berlin, Germany; 3 Harding Center for Risk Literacy, Faculty of Health Sciences Brandenburg, University of Potsdam, Potsdam, Germany; 4 Center for Adaptive Rationality, Max Planck Institute for Human Development, Berlin, Germany; 5 Department of Emergency Medicine, Basel University Hospital, Basel, Switzerland; National Yang-Ming University, TAIWAN

## Abstract

**Background:**

Generalized weakness and fatigue are underexplored symptoms in emergency medicine. Triage tools often underestimate patients presenting to the emergency department (ED) with these nonspecific symptoms (Nemec et al., 2010). At the same time, physicians’ disease severity rating (DSR) on a scale from 0 (not sick at all) to 10 (extremely sick) predicts key outcomes in ED patients (Beglinger et al., 2015; Rohacek et al., 2015). Our goals were (1) to characterize ED patients with weakness and/or fatigue (W|F); to explore (2) to what extent physicians’ DSR at triage can predict five key outcomes in ED patients with W|F; (3) how well DSR performs relative to two commonly used benchmark methods, the Emergency Severity Index (ESI) and the Charlson Comorbidity Index (CCI); (4) to what extent DSR provides predictive information beyond ESI, CCI, or their linear combination, i.e., whether ESI and CCI should be used alone or in combination with DSR; and (5) to what extent ESI, CCI, or their linear combination provide predictive information beyond DSR alone, i.e., whether DSR should be used alone or in combination with ESI and / or CCI.

**Methods:**

Prospective observational study between 2013–2015 (analysis in 2018–2020, study team blinded to hypothesis) conducted at a single center. We study an all-comer cohort of 3,960 patients (48% female patients, median age = 51 years, 94% completed 1-year follow-up). We looked at two primary outcomes (acute morbidity (Bingisser et al., 2017; Weigel et al., 2017) and all-cause 1- year mortality) and three secondary outcomes (in-hospital mortality, hospitalization and transfer to ICU). We assessed the predictive power (i.e., resolution, measured as the Area under the ROC Curve, AUC) of the scores and, using logistic regression, their linear combinations.

**Findings:**

Compared to patients without W|F (n = 3,227), patients with W|F (n = 733) showed higher prevalences for all five outcomes, reported more symptoms across both genders, and received higher DSRs (median = 4; interquartile range (IQR) = 3–6 vs. median = 3; IQR = 2–5). DSR predicted all five outcomes well above chance (i.e., AUCs > ~0.70), similarly well for both patients with and without W|F, and as good as or better than ESI and CCI in patients with and without W|F (except for 1-year mortality where CCI performs better). For acute morbidity, hospitalization, and transfer to ICU there is clear evidence that adding DSR to ESI and/or CCI improves predictions for both patient groups; for 1-year mortality and in-hospital mortality this holds for most, but not all comparisons. Adding ESI and/or CCI to DSR generally did not improve performance or even decreased it.

**Conclusions:**

The use of physicians’ disease severity rating has never been investigated in patients with generalized weakness and fatigue. We show that physicians’ prediction of acute morbidity, mortality, hospitalization, and transfer to ICU through their DSR is also accurate in these patients. Across all patients, DSR is less predictive of acute morbidity for female than male patients, however. Future research should investigate how emergency physicians judge their patients’ clinical state at triage and how this can be improved and used in simple decision aids.

## Introduction

### Background

In the emergency department (ED), decisions have to be made quickly. Misjudgments and misdiagnoses have adverse consequences, especially if resulting in disposition to lower levels of care or to discharge. Therefore, identification of the severely ill is of utmost importance. The use of triage tools is ubiquitous, but these may lack power in certain patient groups, such as older populations [1] and patients with nonspecific complaints such as weakness or fatigue [2]. Weakness and fatigue are associated with higher in-hospital mortality [3], higher resource use, longer ED length of stay, and higher long-term mortality in older patients [4, 5]. However, the characteristics of ED patients with weakness and/or fatigue are not well understood.

ED physicians’ disease severity rating (DSR)—their immediate and subjective judgment of how ill patients look, typically recorded at triage before any other assessment, particularly before receiving test results—is a promising predictor of adverse outcomes. Several studies found that nurses’ and physicians’ DSR can predict mortality in emergency department patients [6]. Another study found that DSR from phlebotomists can outperform the Danish Emergency Process Triage (DEPT) in predicting mortality [7]. Further research has shown that morbidity can be predicted with computerized algorithms based on both clinical markers and physicians’ DSR even in ED patients with nonspecific complaints [8]. When predicting patient outcomes for patients with nonspecific complaints using DSR, physicians’ accuracy was better than chance [9], and morbidity in these patients could be predicted based on DSR [10].

### Objectives

Our goals were (1) to characterize ED patients with weakness and/or fatigue; to explore (2) to what extent physicians’ disease severity rating (DSR) at triage can predict five key outcomes in patients presenting to the ED with or without weakness and/or fatigue (W|F); (3) how well DSR performs relative to two commonly used benchmark methods, the Emergency Severity Index (ESI) and the Charlson Comorbidity Index (CCI); (4) to what extent DSR provides predictive information beyond ESI, CCI, or their linear combination, i.e., whether ESI and CCI should be used alone or in combination with DSR; and (5) to what extent ESI, CCI, or their linear combination provide predictive information beyond DSR alone, i.e., whether DSR should be used alone or in combination with ESI and / or CCI. For the fourth and fifth goal, we use logistic regression models to explore the predictive power of two or three scores when combined into a new score. Importantly, even though logistic regression models give probability values as their output, we interpret those values only as a score and not as a proper probability because it is *not* our goal to already propose prognostic prediction models (e.g., estimated coefficients of a logistic regression model), which then—following best-practices [11]—would need to be thoroughly evaluated with regard to further properties, such as Brier score, calibration curves etc. For all these reasons, we restrict ourselves to reporting ROC curve analyses (see Methods below) and will thus not report Brier scores, calibration curves etc. Nevertheless, to ensure reporting transparency, we adhere to the TRIPOD statement for reporting [11] with respect to the results we do present.

## Methods and materials

### Study design and setting

We carried out a secondary analysis of data that was prospectively collected from October 21 to November 11, 2013, and from February 1 to February 23, 2015, in the ED of a 700-bed urban Swiss academic tertiary care hospital with over 50,000 visits per year [4, 12]. The study protocol was approved by the local ethics committee (236/13, www.eknz.ch). The need to provide written informed consent was waived by the committee. The study was funded out of the research budget of the emergency medicine department at the University Hospital Basel.

### Selection of participants

All patients except obstetric, pediatric, and ophthalmologic patients are seen in the ED. The latter are treated in separate facilities nearby. We recruited patients presenting to the ED 24 hours a day, 7 days a week as part of a quality-control study. All patients presenting to the ED were eligible. The study team asked for verbal consent to participate. Patients were excluded if they actively declined participation or if the electronic health record contained a general rejection to participate in research. Patients who could not be interviewed or give consent because of dementia, intoxication, or severe language barriers were excluded. Patients who required active life-support, were unresponsive, or refused to participate were also excluded. A study team consisting of medical students who were unaware of the purpose of the study was instructed to systematically interview patients, nurses, and physicians. More details on the selection of participants and data collection can be found elsewhere [4].

### Variables / predictors

Triage clinicians were asked the following question at the onset of each triage encounter: ‘How ill does this patient look?’ The disease severity was expressed on an 11-point numeric rating scale (NRS), from 0 (not sick at all) to 10 (extremely sick), with possible values 0, 1, 2, 3, 4, 5, 6, 7, 8, 9, and 10. We asked every patient at presentation whether they suffered from any of the following 35 symptoms: fever, skin rash, headache, dizziness, acute visual disorder, acute hearing disorder, nasal discharge, dysphagia, cough, expectoration, dyspnea, chest pain, abdominal pain, nausea, vomiting, diarrhea, constipation, dysuria, back pain, neck pain, arm pain, leg pain, joint pain, flank pain, joint swelling, leg swelling, altered state of mind, numbness, paralysis, gait disorder, speech disorder, fatigue, weakness, loss of appetite, sleeping disorder. Multiple answers were allowed. The Emergency Severity Index (ESI) score was defined as the urgency level at which a patient needed to be treated according to the German version of the ESI handbook. The Charlson Comorbidity Index (CCI) was calculated for each patient using the ICD-10 based Halfon-Version coding system coded by certified local coders.

Patient demographics (including age, gender and ethnic origin), the ESI triage category and the ICD-10 codes used to calculate the CCI were extracted from the hospital’s electronic health record. The symptoms at presentation including weakness and fatigue, were recorded at the time of inclusion in study case report forms based on systematic interviewing by the study team.

To blind the researchers, all predictors were assessed by trained medical students. Neither nurses nor physicians were aware of the study hypothesis when interviewed by the students immediately after performing the disease severity rating. Instead, they were informed that the reason of the assessment was a quality improvement project.

### Outcomes

We investigated both short-term (acute morbidity, hospitalization, intensive care unit admission, in-hospital mortality) and one long-term outcome (1-year mortality), which were obtained from the electronic health record.

Acute morbidity was defined as any potentially life-threatening condition or any condition requiring early intervention to prevent disability, deterioration, or death [4, 13, 14]. It was assessed based on chart reviews by two physicians and, in case of disagreement, a senior physician who served as a referee. The results were documented on a data abstraction form. A patient was categorized as acutely morbid, if their chart included one or more of the following events: administration of antibiotics, virostatics, antifungals, immunosuppressives, diuretics, anticoagulants, antihypertensives, and procoagulants; the need for invasive interventions, or prolonged monitoring; new neurological deficits, or seizures; fractures, or self-harm. A more complete definition of acute morbidity can be found elsewhere [14].

In-hospital and 1-year mortality were defined as death before discharge from the hospital and death up to 365 days after presentation, respectively. Both were included because while in-hospital mortality is closer related to a patient’s acute medical challenges, 1-year mortality is linked to underlying chronic diseases. Patients were followed up for a year after presentation to assess 1-year mortality. A patients’ clinical condition as well as their emergency physicians’ rating thereof may not be causally related to the outcomes under study such as 1-year mortality. Nevertheless, if the disease severity rating can predict relevant outcomes, it could serve as a red flag for these patients, i.e., it could draw attention to patients who are likely to follow negative health and survival trajectories [15]. Reliable prognoses are an essential component of the practice of emergency medicine. For the individual patient, an unplanned emergency presentation can be a sentinel event with an impact on long-term prognosis [16]. In admitted patients, for example, it was shown that mortality rate was as high as 22% at 1 year. That study also found that admitted patients have an excess risk of dying compared to the background population [17]. Interestingly, although the odds ratio of death was highest in younger patients, the absolute risk of death was greatest in the elderly, suggesting that different disease trajectories come into play. Another study showed how the likelihood of death within the next 12 months is related to male gender, older age, admission to a medical specialty and social deprivation [18]. We have previously studied trajectories of illness in nonspecific complaints [15] and developed a framework containing five categories (functional, therapy-induced, deterioration of chronic condition, acute new condition, and acute event in a chronic condition). We found that each category has a distinct trajectory, as expressed by survival curves, thereby assisting prognostication. A related study found that certain factors, such as acute infections, were associated with unfavorable prognosis in patients with advanced dementia studied over the course of 2 years [19].

In summary, there is a strong need for hospitals to adopt a more evidence-based approach to identify patients who are entering the last year of their lives (i.e., 1 year follow-up). This prognostic information is needed in order to make and deliver appropriate care plans. Once entered into a patient’s electronic health record, the emergency physicians’ disease severity rating could be informative for a patient’s healthcare providers beyond their treating emergency physician.

Hospitalization was defined as admission to any hospital in-patient department, including disposition to other acute care hospitals directly from the emergency department within 24 hours. Intensive care unit admission was defined as admission to one of the hospital’s medical or surgical intensive care units, intermediate care units, or into stroke or neurosurgical intensive care during the index hospitalization (the hospitalization that we analyzed the data from).

The following outcomes were assessed in a blinded fashion using database matching: in-hospital mortality, 30-day mortality, hospitalization, and intensive care.

### Analyses

The analyses describing the population included the data of all patients. One-year mortality was missing for 227 patients. Therefore, these patients were excluded when analyzing 1-year mortality as an outcome. For the Receiver Operating Characteristics (ROC) curve analyses and the logistic regression models (see below), 49 patients were excluded because they had missing data on the disease severity rating (DSR; 43 missing) or the Emergency Severity Index (6 missing).

In all analyses patients with weakness and/or fatigue were compared with patients with neither weakness nor fatigue. To describe the patient population, patients were additionally split by gender for some analyses.

The predictive power of the three scores—disease severity rating (DSR), Emergency Severity Index (ESI), and, Charlson Comorbidity Index (CCI)—and their linear combinations were quantified using Receiver Operating Characteristics (ROC) curves [20], which plot for all possible thresholding values of a score the resulting sensitivity and specificity with respect to the outcome. The Area Under the ROC Curve (AUC) is a common metric to summarize the resolution (aka discrimination ability) of a score across all possible thresholding values. An AUC value can be interpreted as follows: If one patient with and one patient without an outcome (e.g., acute morbidity) is each randomly sampled from the corresponding group of patients, then the AUC value indicates the probability that the patient with the outcome has a higher score. In other words, AUC values represent the probability that a score correctly ranks patients according to their risk of experiencing an outcome.

We calculated AUC using the R package **pROC** v1.16.2 [21] using the trapezoid method (i.e., no smoothing); this method allows interpreting AUC values in terms of the probability of correctly ranking patients as discussed above. Furthermore, the direction of the scores (i.e., whether increasing or decreasing values indicate higher risk) was fixed (i.e., not estimated from the data) to avoid optimistic bias in the cross-validation analyses (see below).

When evaluating the three scores on their own, we calculated the AUC in-sample and report 95% confidence intervals (calculated using the DeLong method implemented in **pROC**). Since the score values are evaluated directly (i.e., no logistic regression models are used), there are no free parameters that are fit to the data; thus evaluating the performance in-sample is appropriate (i.e., no danger of overfitting).

When evaluating the performance of combinations of scores, we used standard logistic regression models (using the function *glm* with the family *binomial(link = “logit”)* in the **stats** R base package v4.0.2). To avoid overfitting the training data, we calculated AUC out-of-sample using 10-times repeated 5-fold cross-validation [22]. We report the mean AUC across validation folds and an approximate 95% confidence interval (calculated as mean AUC ±1.96·SD/√k, where SD is the sample standard deviation of the AUC values across the validation folds and *k* is the number of folds per CV repeat, here *k =* 5).

## Results

### Characteristics of study subjects

**Fig 1:**
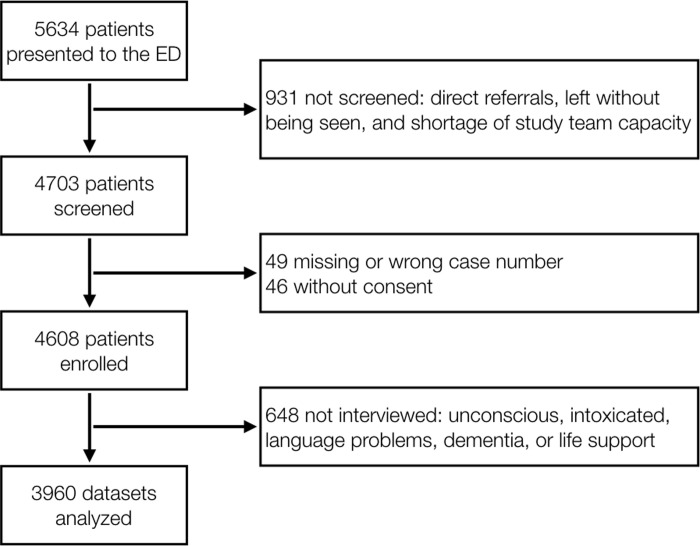
Inclusion flowchart. Adapted from Bingisser et al., *European Journal of Internal Medicine*, 2017.

Sample size was not prospectively planned and was based on 5,634 presentations. In sum, 3,960 patients were included in the study (Fig 1).

**Fig 2:**
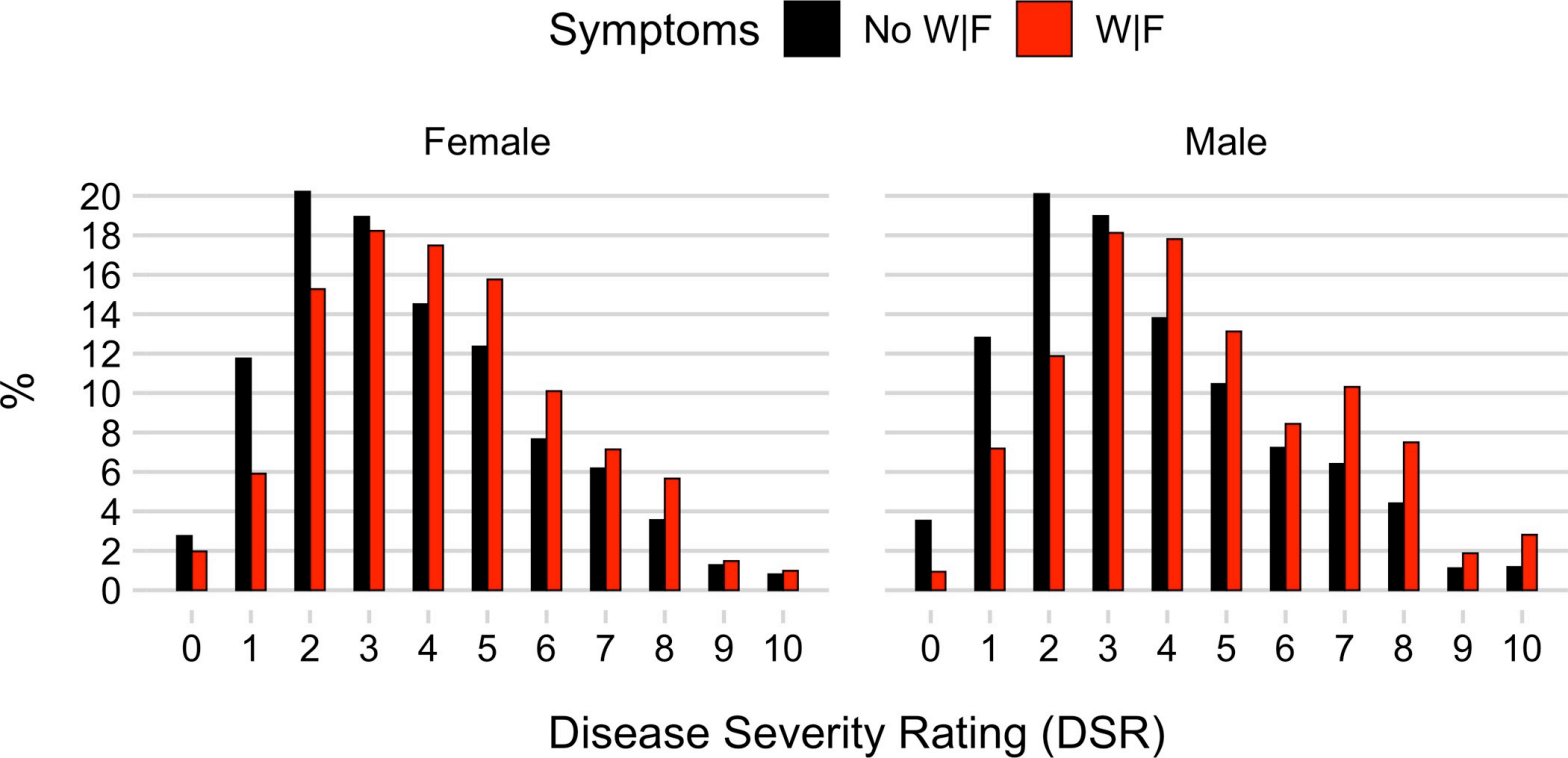
Histogram of the physicians’ disease severity rating for all-comers (n = 3,917), separately females and males and patients with and without weakness and/or fatigue. Counts are normalized to percentages, separately for each of the four subgroups.

Table 1 describes the study subjects’ characteristics, which are also reported elsewhere [4]. Patients presenting with W|F were more likely to be female (56%) than patients without these symptoms (47%), and they also had a higher median age of 55 years (IQR = 35–74) as compared to patients without W|F, whose median age was 49 (IQR 32–71). This age difference was more pronounced in male (58 years vs. 49 years) than in female patients (53 years vs. 50 years). While the median age in patients with neither weakness nor fatigue was similar in male (49, IQR = 32–69) and female patients (50, IQR = 33–73), females with W|F (median = 53, IQR = 35–73) were somewhat younger than males with either symptom (median = 58, IQR = 36–74). Male patients and female patients with W|F reported more symptoms than those without (median = 4 [IQR = 3–6] vs. 1 [IQR = 1–2] in males and median = 5 [IQR = 3–7] vs. 1 [IQR = 1–3] in females). In all four groups, the median ESI category was 3, with an IQR of 2–3 in patients with W|F and an IQR of 3–4 in patients without. In both males and females with W|F, the main other symptoms were dizziness and headache. Both genders with neither weakness nor fatigue most commonly reported leg pain and headache. Both genders with W|F were transferred to the ICU (males = 9% and females = 7%) more often than those without W|F (males = 6% and females = 4%). Based on their disease severity rating (Fig 2), ED physicians rated patients with weakness or fatigue as sicker (median = 4, IQR = 3–6) than all other patients (median = 3, IQR = 2–5).

**Table 1:** Baseline characteristics study population (n = 3960).

Patients	Weakness and/or fatigue		No weakness or fatigue		Total
	Male	Female	Male	Female	
Cases, n (%)	324 (8)	409 (10)	1724 (44)	1503 (38)	3960 (100)
Age years, median (IQR)	58 (36–74)	53 (35–73)	49 (32–69)	50 (33–73)	51 (33–71)
Ethnic origin, n (%)					
Central/Northern Europe	224 (69)	279 (68)	1152 (67)	1072 (70)	2727 (69)
Mediterranean	29 (9)	40 (10)	179 (10)	107 (7)	355 (9)
Southeastern Europe	25 (8)	26 (6)	88 (5)	82 (5)	221 (6)
Rest of Eastern Europe	9 (3)	11 (3)	70 (4)	44 (3)	134 (3)
Turkey	21 (6)	21 (5)	102 (6)	89 (6)	233 (6)
Africa	7 (2)	11 (3)	51 (3)	24 (2)	93 (2)
Asia	7 (2)	13 (3)	55 (3)	50 (3)	125 (3)
North America/Australia	1 (0.3)	1 (0.2)	9 (0.5)	9 (1)	20 (1)
Central/South America	1 (0.3)	7 (2)	14 (0.8)	16 (1)	38 (1)
NA	0	0	4 (0.2)	10 (1)	14 (0)
ESI category, n (%)					
1	3 (1)	4 (1)	26 (2)	19 (1)	52 (1)
2	111 (34)	102 (25)	352 (20)	263 (18)	828 (21)
3	129 (40)	213 (52)	620 (36)	576 (38)	1538 (39)
4	79 (24)	89 (22)	655 (38)	582 (39)	1405 (35)
5	1 (0.3)	1 (0.2)	68 (4)	61 (4)	131 (3)
NA	1 (0.3)	0	3 (0.2)	2 (0.1)	6 (0)
ESI: 1	3 (1)	4 (1)	26 (2)	19 (1)	52 (1)
ESI: 2	111 (34)	102 (25)	352 (20)	263 (17)	828 (21)
ESI: 3	129 (40)	213 (52)	620 (36)	576 (38)	1538 (39)
ESI: 4	79 (24)	89 (22)	655 (38)	582 (39)	1405 (35)
ESI: 5	1 (0)	1 (0)	68 (4)	61 (4)	131 (3)
ESI: NA	1 (0)	0 (0)	3 (0)	2 (0)	6 (0)
CCI: 0	247 (76)	330 (81)	1498 (87)	1322 (88)	3397 (86)
CCI: 1	21 (6)	29 (7)	78 (5)	77 (5)	205 (5)
CCI: 2	19 (6)	28 (7)	72 (4)	53 (4)	172 (4)
CCI: 3	13 (4)	9 (2)	27 (2)	23 (2)	72 (2)
CCI: 4	8 (2)	7 (2)	22 (1)	14 (1)	51 (1)
CCI: 5+	16 (5)	6 (1)	27 (2)	14 (1)	63 (2)
DSR: 0	3 (1)	8 (2)	60 (3)	41 (3)	112 (3)
DSR: 1	23 (7)	24 (6)	218 (13)	175 (12)	440 (11)
DSR: 2	38 (12)	62 (15)	342 (20)	301 (20)	743 (19)
DSR: 3	58 (18)	74 (18)	323 (19)	282 (19)	737 (19)
DSR: 4	57 (18)	71 (17)	235 (14)	216 (14)	579 (15)
Number of symptoms, median (IQR)	4 (3–6)	5 (3–7)	1 (1–2)	1 (1–3)	
Most common symptoms, n (%)					
	Weakness	Weakness	Leg pain	Headache	
	238 (73)	318 (78)	276 (16)	247 (16)	
	Fatigue	Fatigue	Headache	Leg pain	
	188 (58)	264 (65)	218 (13)	240 (16)	
	Dizziness	Dizziness	Arm pain	Arm pain	
	98 (30)	154 (38)	215 (12)	189 (13)	
	Headache	Headache	Back pain	Dizziness	
	91 (28)	151 (37)	193 (11)	187 (12)	
	Cough	Nausea	Chest pain	Abdominal pain	
	72 (22)	113 (28)	189 (11)	180 (12)	

IQR = Interquartile range; ESI = Emergency Severity Index; CCI = Charlson Comorbidity Index. All percentages in brackets are column percentages except the ones for cases, which are row percentages.

Patients’ 1-year mortality was not recorded in 227 cases (5.7%). To investigate whether these patients’ characteristics differed from the rest, we compare the two groups in S1 Table. Patients with missing values were on average 14 years younger and were slightly more likely to have an ESI score of 4 or higher but did not differ from other patients in any noticeable way.

### Characterizing ED patients with weakness and/or fatigue

Table 2 shows the outcomes for patients with and without W|F, split by gender. Sixty-nine percent of all patients were treated as outpatients. The median length of stay (LOS) of inpatients was 5 days. Forty percent of all patients suffered from acute morbidity, 6% were transferred to the ICU, 1% died in hospital, and 5% died within a year. One-year follow-up was completed for 3,733 patients (94%). Patients with W|F (n = 733) differed from all other patients: Acute morbidity was found in 45% of patients with W|F and in 38% of all other patients. One-year mortality was 8% in patients with W|F and 5% in all other patients. In-hospital mortality was 2% in patients with W|F and 1% in all other patients. Hospitalization rate was 42% in patients with W|F and 29% in all other patients. Intensive care use was 8% in patients with W|F and 5% in all other patients.

**Table 2:** Outcomes study population.

	Weakness and/or fatigue		No weakness or fatigue	
Patients	Male	Female	Male	Female
	n = 324	n = 409	n = 1724	n = 1503
Acute morbidity, n (%)	165 (51)	163 (40)	652 (38)	585 (39)
1-year mortality n (%)	36 (11)	25 (6)	92 (5)	62 (4)
In-hospital mortality, n (%)	9 (3)	7 (2)	20 (1)	19 (1)
Hospitalization, n (%)	149 (46)	156 (38)	486 (28)	446 (30)
Transferred to ICU, n (%)	29 (9)	30 (7)	95 (6)	65 (4)

### To what extent can physicians’ disease severity rating (DSR) predict key outcomes in patients with or without weakness and/or fatigue?

**Fig 3:**
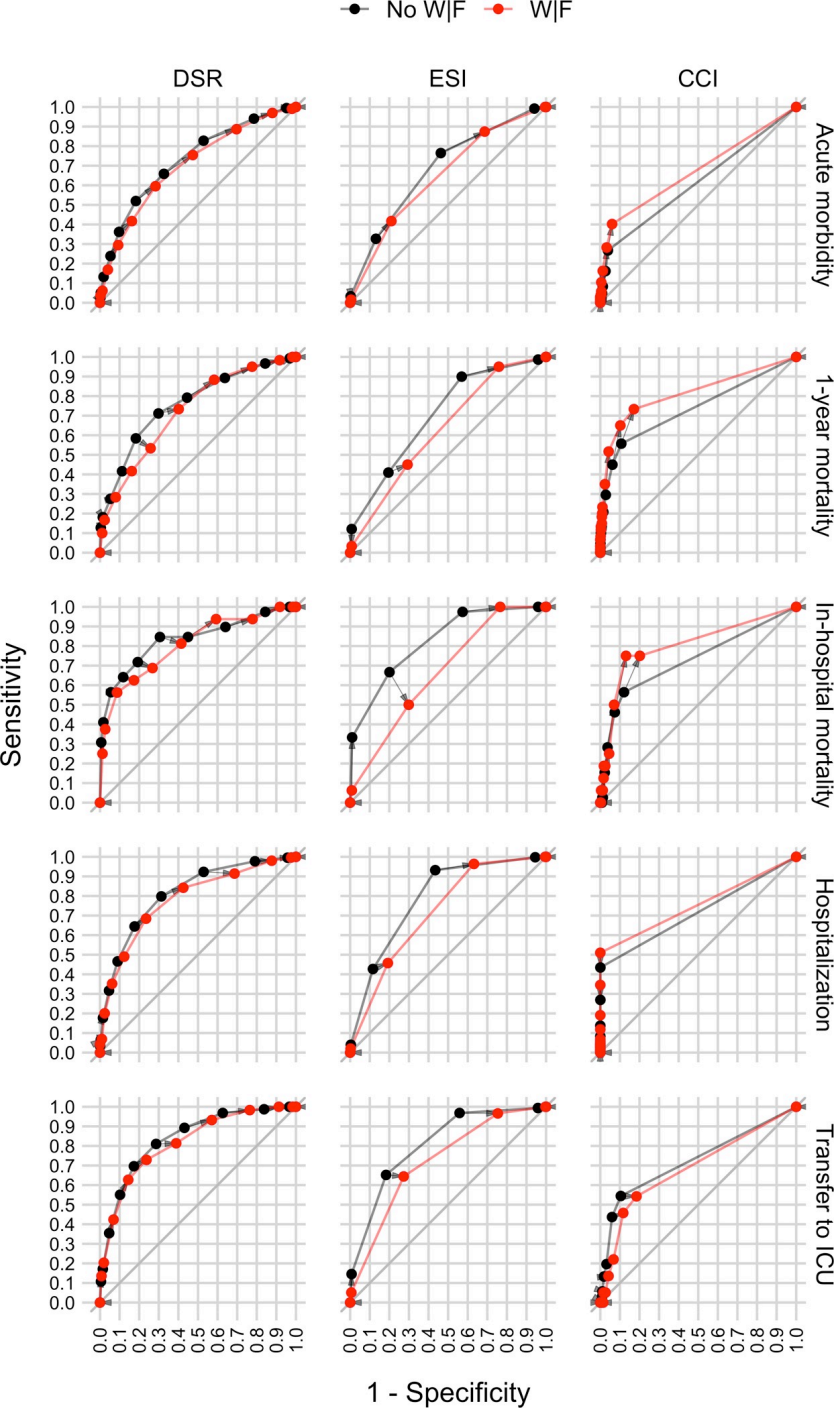
Receiver Operating Characteristics (ROC) curves showing the discrimination ability of three ED triaging scores to identify ED patients at risk for five different outcomes, separately for patients with or without weakness and/or fatigue (W|F). Panel columns show results for the three scores: Disease severity rating (DSR), Emergency Severity Index (ESI), and Charlson Comorbidity Index (CCI). Panel rows show results for the five outcomes: Acute morbidity, 1-year mortality, in-hospital mortality, hospitalization, and transfer to ICU. Each panel shows two ROC curves for a particular combination of score and outcome: One ROC curve for patients with W|F (“W|F” in red) and one ROC curve for patients with other symptoms (“No W|F” in black). Each point shows the sensitivity (y-axis) and 1 –specificity (x-axis) for each possible thresholding value for a score (i.e., possible ROC operating points). The corresponding operating points for patients with and without W|F are connected by an arrow, which highlights how, if at all, the sensitivity and specificity of the same thresholding value for a particular score differs between the two patient groups.

**Fig 4:**
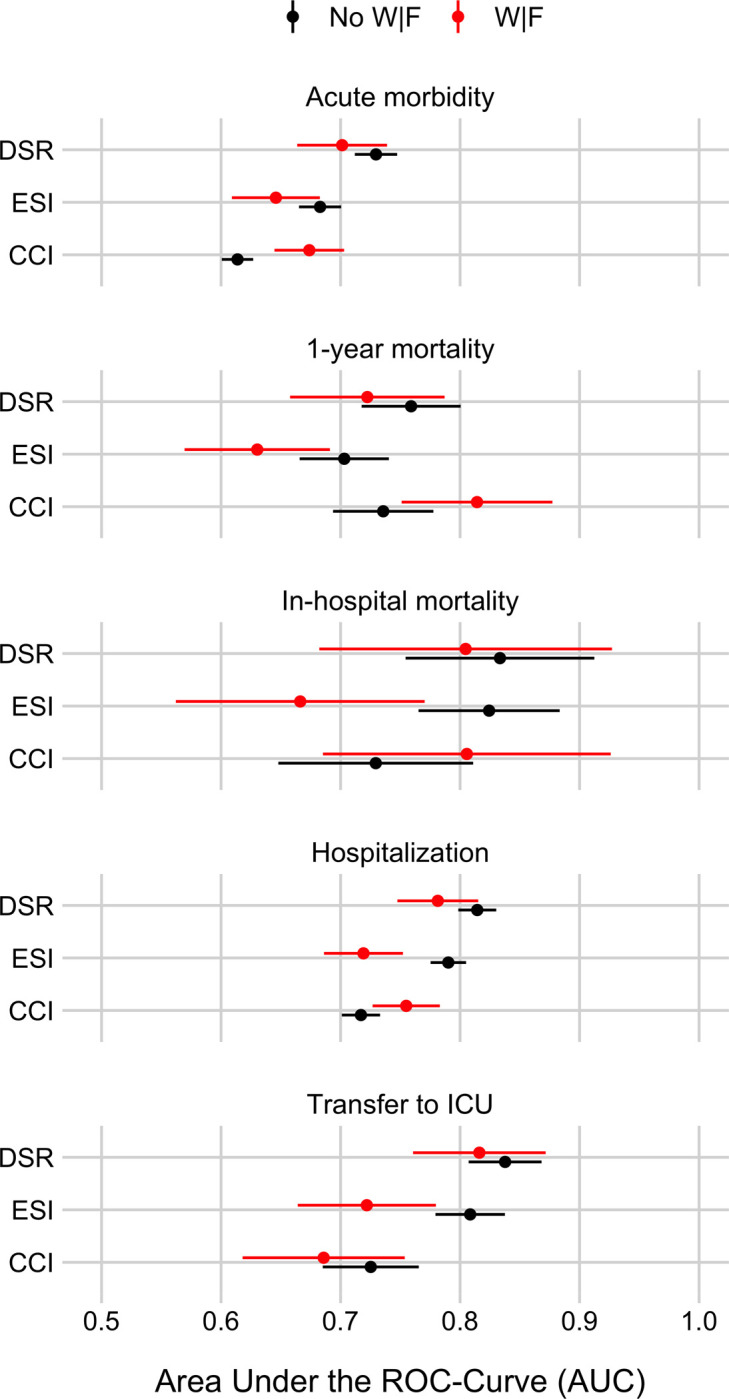
Area Under the Receiver Operating Characteristics (ROC) curves (AUC) summarizing the discrimination ability (resolution) of the three ED triaging scores to identify ED patients at risk for five different outcomes, separately for patients with or without weakness and/or fatigue (W|F). Panel rows show results for the five outcomes: Acute morbidity, 1-year mortality, in-hospital mortality, hospitalization, and transfer to ICU. Each panel shows the in-sample AUC values (plus a 95% confidence interval, CI) for the three scores, disease severity rating (DSR), Emergency Severity Index (ESI), and Charlson Comorbidity Index (CCI), separately for patients with W|F (in red) and for patients with other symptoms (“No W|F” in black). See Methods for details on how AUC and CI were calculated.

Figs 3 and 4 show the extent to which DSR, the Emergency Severity Index (ESI), and the Charlson Comorbidity Index (CCI) can predict the five outcomes, separately for patients with and without weakness and/or fatigue (W|F). Fig 3 shows Receiver Operating Characteristics (ROC) curves and Fig 4 shows the Areas Under those ROC curves (AUC; Fig 4). Results show that DSR can predict all five outcomes for both patient groups well above chance (i.e., AUCs > ~0.70). Furthermore, there is no clear evidence that DSR performs worse for patients with W|F.

### How well does the Disease Severity Rating (DSR) perform compared to two commonly used benchmark scores?

Figs 3 and 4 show that DSR predicts patient outcomes as good as or better than ESI and CCI. The same pattern holds also when separately comparing the scores for patients with and without weakness and/or fatigue (W|F) with one exception: There is some evidence that for patients with W|F CCI predicts 1-year mortality better than DSR.

The above analyses did not adjust for typical covariates, such as age and sex. For a complete picture, we contrast the predictive power of DSR vis-á-vis age and sex to gauge DSR’s predictive power beyond those two standard demographic variables. S1 Fig shows how DSR and age relate to each other, separately for female and male patients with or without symptoms of weakness or fatigue. The figure shows that DSR increases with older age, but there is clear excess variance not explained by age. Additionally, to check whether DSR predicts the outcomes beyond a control model that accounts for age and sex, we compared two additional models (*age + sex*, *DSR + age + sex*) to the DSR-only model and report this comparison in S2 Fig. Results show that DSR clearly provides non-redundant predictive information beyond age and sex for acute morbidity, hospitalization, and transfer to ICU (i.e., the *DSR + age + sex* model outperforms the *age + sex* model). In contrast, for 1-year mortality and in-hospital mortality the combined model (*DSR + age + sex*) does not clearly perform better than the control model (*age + sex*).

### To what extent does the Disease Severity Rating (DSR) provide predictive information beyond the two benchmark scores or their linear combination?

**Fig 5:**
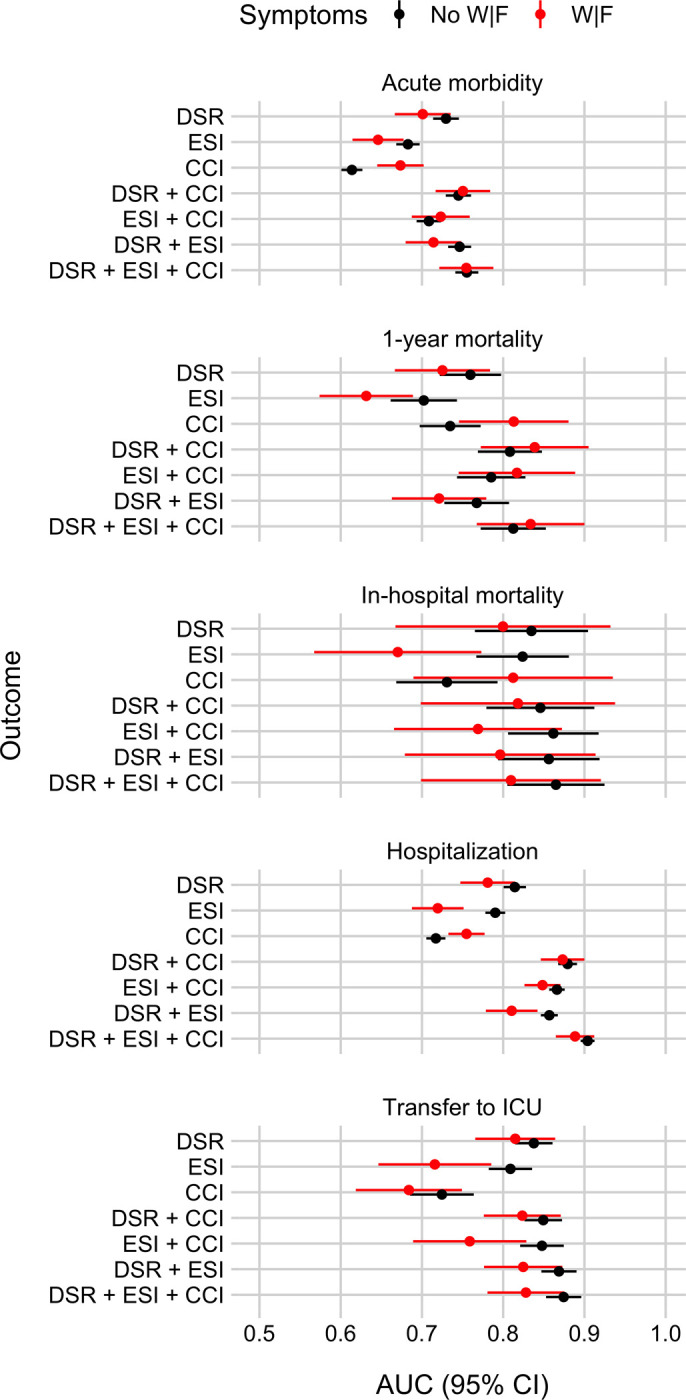
Areas Under the Receiver Operating Characteristics curve (AUC) summarizing the discrimination ability of three ED triaging scores and combinations of them to identify ED patients at risk for five different outcomes, separately for patients with or without weakness and/or fatigue (W|F). Panel rows show results for the five outcomes: Acute morbidity, 1-year mortality, in-hospital mortality, hospitalization, and transfer to ICU. Each panel shows cross-validated AUC values (plus a 95% confidence interval, CI) for the three scores and linear combinations of them, separately for patients with W|F (in red) and for patients with other symptoms (“No W|F” in black). See Methods for details on how AUC and CI were calculated.

**Fig 6:**
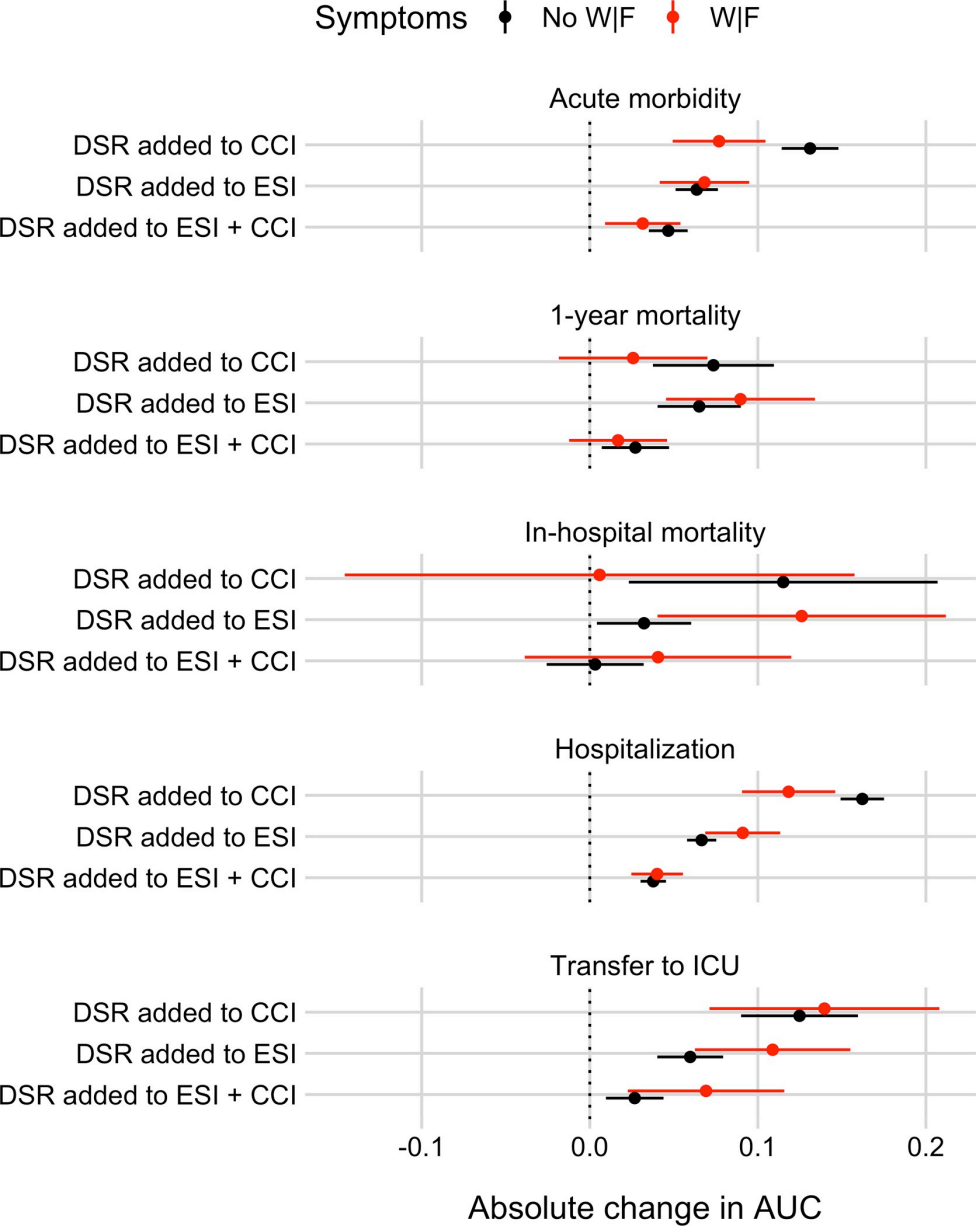
Absolute change in Area Under the Receiver Operating Characteristics curves (AUC) for identifying ED patients at risk for five different outcomes when adding the Disease Severity Rating (DSR) to either the Emergency Severity Index (ESI), the Charlson Comorbidity Index (CCI), or both, separately for patients with or without weakness and/or fatigue (W|F). Panel rows show results for the five outcomes: Acute morbidity, 1-year mortality, in-hospital mortality, hospitalization, and transfer to ICU. Each panel shows cross-validated absolute changes in AUC (plus a 95% confidence interval, CI), separately for patients with W|F (in red) and for patients with other symptoms (“No W|F” in black). See Methods for details on how AUC and CI were calculated.

Fig 5 shows the extent to which disease severity rating (DSR), the Emergency Severity Index (ESI), the Charlson Comorbidity Index (CCI), and all linear combinations of two or three scores can predict the five outcomes, separately for patients with and without weakness and/or fatigue (W|F). Fig 6 shows absolute changes in the AUC when adding DSR to ESI, CCI, or a linear combination of ESI and CCI. For acute morbidity, hospitalization, and transfer to ICU there is clear evidence that DSR improves predictions for both patients with and without W|F; for 1-year mortality and in-hospital mortality this result holds for most, but not all comparisons. Consistent with the above-reported result that CCI performs better than DSR in predicting 1-year mortality in patients with W|F, adding DSR to CCI does not clearly improve performance. Because of the low prevalence of in-hospital mortality in the current population (1–3%; see Table 2), the results for this outcome come with considerable uncertainty. Having said that, there is some evidence that DSR improves on ESI, but not on CCI or a combination of ESI and CCI.

### To what extent do the two benchmark scores or their linear combination provide predictive information beyond the Disease Severity Rating (DSR) alone?

**Fig 7:**
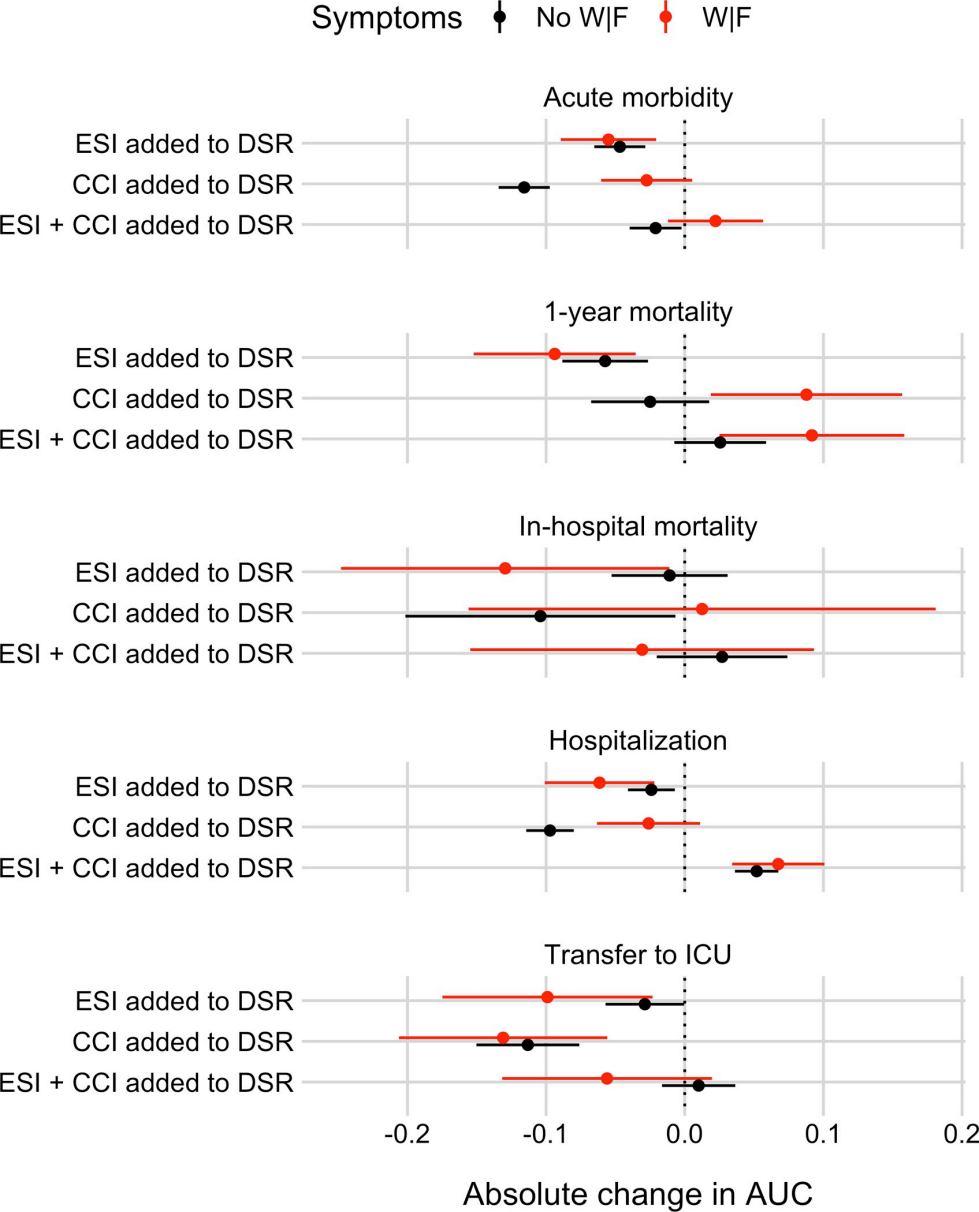
Absolute change in Area Under the Receiver Operating Characteristics curves (AUC) for identifying ED patients at risk for five different outcomes when adding the Emergency Severity Index (ESI), the Charlson Comorbidity Index (CCI), or both to the disease severity rating (DSR), separately for patients with or without weakness and/or fatigue (W|F). Panel rows show results for the five outcomes: Acute morbidity, 1-year mortality, in-hospital mortality, hospitalization, and transfer to ICU. Each panel shows cross-validated absolute changes in AUC (plus a 95% confidence interval, CI), separately for patients with W|F (in red) and for patients with other symptoms (“No W|F” in black). See Methods for details on how AUC and CI were calculated.

Fig 7 shows absolute changes in the AUC when adding the Emergency Severity Index (ESI), the Charlson Comorbidity Index (CCI), or their linear combination to DSR. The results show that generally adding one or both benchmark scores to DSR does not improve performance or even decrease it, with two exceptions. First, consistent with the above-reported results on 1-year mortality, adding CCI to DSR does improve performance for patients with weakness and/or fatigue (W|F). Second, for hospitalization, adding ESI and CCI to DSR improves performance for both patient groups; however, only adding ESI or CCI to DSR does not.

## Discussion

Little is known about patients who present to the ED with weakness and/or fatigue (W|F). To shed light on these patients, we characterized this patient group by comparing demographics and outcomes with an all-comer cohort. Previous research has shown that physicians’ disease severity rating (DSR) can predict key outcomes in ED patients [10, 12]. We investigated whether physicians’ DSR of patients with W|F, elicited at the beginning of triage, predicts five key patient outcomes: acute morbidity, 1-year mortality, in-hospital mortality, hospitalization, and transfer to the ICU. We further compared the predictive power of DSR (in terms of Area Under the ROC Curve, AUC) with that of two established benchmarks—the Emergency Severity Index (ESI) and the Charlson Comorbidity Index (CCI). Patients presenting to the ED with W|F were older, had a higher number of symptoms, and had worse outcomes than all other patients. Overall, physicians’ DSR was predictive of all five outcomes for both patients with and without W|F. Compared to ESI and CCI, two widely used and established scores, the disease severity rating, a very frugal assessment tool, was generally as or even more predictive and generally added predictive information beyond the other two scores. For acute morbidity, in-hospital mortality, and transfer to ICU, there is no clear evidence that adding ESI and/or CCI to DSR improves predictive performance. This suggests that for these outcomes DSR might be sufficient, and could be used for informal triage. However, to assess the longer-term trajectory of ED patients in terms of their 1-year mortality, our results suggest that combining DSR with CCI is more predictive than DSR alone. Similarly, as reported in S2 Fig, adding the DSR to a linear model predicting 1-year mortality based on age and sex does not yield better predictions than the combination of only age and sex.

Given that triage sometimes fails female patients at a higher rate than male patients, we also explored possible gender differences in the disease severity rating’s (DSR) ability to predict the five outcomes in this study. S3 and S4 Figs in the supporting information show that DSR is similarly predictive for 1-year mortality, in-hospital mortality, and transfer to ICU, whereas it is somewhat less predictive for women for acute morbidity and hospitalization. Importantly, however, even in those two cases DSR performs similarly or even better than the two benchmark scores.

These results merit discussion: First, demographics of patients presenting with weakness and/or fatigue (W|F) differ from those of patients not presenting with either or both of those two symptoms. It is a common belief that nonspecific symptoms (weakness and fatigue being the most common) seem to occur more frequently in the older population [5]. However, the present all-comer study demonstrates that age differences are not as pronounced as one might have anticipated.

We can only speculate about the reasons for the gender-related difference in how well DSR can predict acute morbidity. Differences in presenting symptoms have previously been shown: women generally present with more symptoms than men [13] and tend to present with other symptoms in acute diseases, such as myocardial infarction [23]. Finally, female patients have a higher morbidity in nonspecific presentations [24]. Cultural norms and biases with respect to gender could influence both patient expression of symptoms as well as physicians’ perception of disease severity. It follows that DSR can be expected to be influenced by cultural norms and provider assumptions and cultural biases. This may limit generalizability across systems and cultures.

Second, the fact that patients with W|F are polysymptomatic is not a new finding, but has not been described in a prospective all-comer population. The reason for the high number of symptoms at presentation—even higher in female patients than in male—is unknown, but could not be attributed to polymorbidity in a recent analysis [13].

Third, the outcomes in ED patients with W|F are worse regarding morbidity and 1-year mortality. This has already been shown [4]; however, the generally comparable outcomes in both genders in W|F patients are a new finding. Because these unfavorable outcomes are difficult to foresee, triage systems relying on typical case vignettes are weak in assessing nonspecific complaints. We investigated whether DSR elicited at triage could be used to predict key outcomes in patients with W|F, a group that is difficult to assess at triage. While DSR has been shown to predict morbidity in an all-comer population [12] and mortality in a group of older patients with nonspecific complaints [10], its use for patients with W|F was not considered before our study, which showed that DSR is as predictive for these ED patients as it is for ED patients with other symptoms. DSR predicts morbidity and hospitalization worse for female compared to male patients—yet another effect of gender that is difficult to interpret. As pointed out above, female patients also tend to report more symptoms which is associated with a higher use of resources and a longer length of stay in the emergency department [4, 13]. Underestimating morbidity could lead to detrimental consequences such as undertreatment, whereas overestimating morbidity may lead to inefficient use of resources. Therefore, judgments based on clinical impression should be used with caution, and further research is needed to understand how emergency physicians judge their patients’ clinical state at triage. This question could be addressed with the tools of cognitive data science by modeling physicians’ judgments with patients’ characteristics. Once we understand this process in more detail, interventions could be developed to improve physicians’ disease severity rating through training.

This study carries some limitations. It is a single center study carried out in Switzerland, and the results therefore cannot be broadly generalized. Although patients with W|F are also common in other populations [2, 25–27], it is unclear to what extent cultural or genetic differences need to be considered when generalizing our results to other regions or countries; judging how ill patients look on a numeric rating scale has not been validated with outcome data in economically, culturally or genetically different populations. However, the alignment of DSR with patients’ morbidity indicates construct validity, the consensus between physicians and nurses indicates inter-rater reliability [12], and the similar patterns in the present data set and the data set of patients with nonspecific complaints described in [2] indicates external validity.

In-hospital mortality, 1-year mortality and also transfers to the ICU were relatively rare events (depending on the subgroup, between 1–3%, 4–9%, and 4–11%), which is why statistical inferences about these outcomes are less certain. The other outcomes, however, occurred at much higher rates (28% or higher).

Finally, decision aids based on regression models (e.g., logistic regression models expressed as nomograms [28]) require computations that cannot simply be executed by physicians and can, therefore, only be implemented into electronic decision support systems. Since combining physicians’ DSR with ESI or CCI can improve on the individual scores for some outcomes, future research should investigate how these predictors can be combined into a user-friendly prediction tool, for example, using fast-and-frugal decision trees [29, 30], which can be implemented into clinical practice at very little cost as pocket cards or posters.

## Conclusions

We show that physicians’ prediction of acute morbidity, mortality, hospitalization, and transfer to ICU through their disease severity rating (DSR) is also accurate in patients who present to the emergency department with generalized weakness and/or fatigue. Across all patients, DSR is less predictive of acute morbidity for female than male patients, however. Future research should investigate how emergency physicians judge their patients’ clinical state at triage and how this can be improved and used in simple decision aids.

## Supporting information

S1 Table Baseline characteristics study population (n = 3960), separated for patients with (TRUE) vs. without (FALSE) NAs in 1-year mortality. (PDF)

S1 Fig This figure shows how DSR and age relate to each other, separately for female and male patients with or without weakness or fatigue. DSR increases with older age, but there is clear excess variance not explained by age. The blue lines and the grey bands show smoothed conditional means and their pointwise 95% confidence interval, respectively, based on generalized additive models.
(TIFF)

S2 Fig Areas Under the Receiver Operating Characteristics curve (AUC) summarizing the discrimination ability of the Disease Severity Score (DSR) compared to age and sex as well as combinations of them to identify ED patients at risk for five different outcomes, separately for patients with or without weakness and/or fatigue (W|F). Panel rows show results for the five outcomes: Acute morbidity, 1-year mortality, in-hospital mortality, hospitalization, and transfer to ICU. Each panel shows cross-validated AUC values (plus a 95% confidence interval, CI) for DSR, age, and sex, separately for patients with W|F (in red) and for patients with other symptoms (“No W|F” in black). See Methods for details on how AUC and CI were calculated.
(TIFF)

S3 Fig Receiver Operating Characteristics (ROC) curves showing the discrimination ability of three ED triaging scores to identify ED patients at risk for five different outcomes, separately for female and male patients. Panel columns show results for the three scores: Disease Severity Rating (DSR), Emergency Severity Index (ESI), and Charlson Comorbidity Index (CCI). Panel rows show results for the five outcomes: Acute morbidity, 1-year mortality, in-hospital mortality, hospitalization, and transfer to ICU. Each panel shows two ROC curves for a particular combination of score and outcome: One ROC curve for female patients (in red) and one ROC curve for male patients (in black). Each point shows the sensitivity (y-axis) and 1 –specificity (x-axis) for each possible thresholding value for a score (i.e., possible ROC operating points). The corresponding operating points for female and male patients are connected by an arrow, which highlights how, if at all, the sensitivity and specificity of the same thresholding value for a particular score differs between the two patient groups.
(TIFF)

S4 Fig Area Under the Receiver Operating Characteristics curves (AUC) summarizing the discrimination ability (resolution) of the three ED triaging scores to identify ED patients at risk for five different outcomes, separately for female and male patients. Panel rows show results for the five outcomes: Acute morbidity, 1-year mortality, in-hospital mortality, hospitalization, and transfer to ICU. Each panel shows the in-sample AUC values (plus a 95% confidence interval, CI) for the three scores, Disease Severity Rating (DSR), Emergency Severity Index (ESI), and Charlson Comorbidity Index (CCI), separately for female patients (in red) and male patients (in black). See Methods for details on how AUC and CI were calculated.
(TIFF)
