# Supplementary material for: Emergency department patients with weakness or fatigue: Can physicians predict their outcomes at the front door? A prospective observational study
Source: PLoS One. 2020 Nov 5;15(11):e0239902. doi: 10.1371/journal.pone.0239902 (PMC7643999; doi:10.1371/journal.pone.0239902)
Supplement: S1 Table — (PDF) [file pone.0239902.s001.pdf]

# Supporting information

**Table S1.** Baseline characteristics study population (n = 3960), separated for patients with (`TRUE`) vs. without (`FALSE`) NAs in 1-year mortality.

| Patients | Missings in 1-year mortality | |
|---|---|---|
| | FALSE | TRUE |
| Cases, n (%) | 3733 (100) | 227 (100) |
| Age years, median (IQR) | 52 (33-72) | 38 (29-53) |
| Ethnic origin, n (%) | | |
| Central/Northern Europe | 2607 (70) | 120 (53) |
| Mediterranean | 327 (9) | 28 (12) |
| Southeastern Europe | 206 (6) | 15 (7) |
| Rest of Eastern Europe | 119 (3) | 15 (7) |
| Turkey | 228 (6) | 5 (2) |
| Africa | 82 (2) | 11 (5) |
| Asia | 104 (3) | 21 (9) |
| North America/Australia | 16 (0) | 4 (2) |
| Central/South America | 33 (1) | 5 (2) |
| NA | 11 (0) | 3 (1) |
| ESI category, n (%) | | |
| 1 | 47 (1) | 5 (2) |
| 2 | 784 (21) | 44 (19) |
| 3 | 1475 (40) | 63 (28) |
| 4 | 1301 (35) | 104 (46) |
| 5 | 120 (3) | 11 (5) |
| NA | 6 (0) | NA |
| CCI: 0 | 3187 (85) | 210 (93) |
| CCI: 1 | 196 (5) | 9 (4) |
| CCI: 2 | 167 (4) | 5 (2) |
| CCI: 3 | 71 (2) | 1 (0) |
| CCI: 4 | 50 (1) | 1 (0) |
| CCI: 5+ | 62 (2) | 1 (0) |
| DSR: 0 | 101 (3) | 11 (5) |
| DSR: 1 | 404 (11) | 36 (16) |
| DSR: 2 | 688 (18) | 55 (24) |
| DSR: 3 | 691 (19) | 46 (20) |
| DSR: 4 | 551 (15) | 28 (12) |
| DSR: 5 | 451 (12) | 17 (7) |
| DSR: 6 | 293 (8) | 12 (5) |
| DSR: 7 | 252 (7) | 11 (5) |
| DSR: 8 | 169 (5) | 6 (3) |
| DSR: 9 | 49 (1) | 1 (0) |
| DSR: 10 | 43 (1) | 2 (1) |
| Number of symptoms, median (IQR) | 2 (1-3) | 1 (1-3) |
| Most common symptoms, n (%) | | |
| | Headache: 657 (18) | Headache: 50 (22) |

| | |
|---|---|
| Leg pain: 593 (16) | Leg pain: 34 (15) |
| Dizziness: 576 (15) | Dizziness: 33 (15) |
| Weakness: 535 (14) | Back pain: 30 (13) |
| Back pain: 480 (13) | Abdominal pain: 29 (13) |

IQR = Interquartile range; ESI = Emergency Severity Index; CCI = Charlson Comorbidity Index
