# Supplementary figures and images for: Emergency department patients with weakness or fatigue: Can physicians predict their outcomes at the front door? A prospective observational study
Source: PLoS One. 2020 Nov 5;15(11):e0239902. doi: 10.1371/journal.pone.0239902 (PMC7643999; doi:10.1371/journal.pone.0239902)

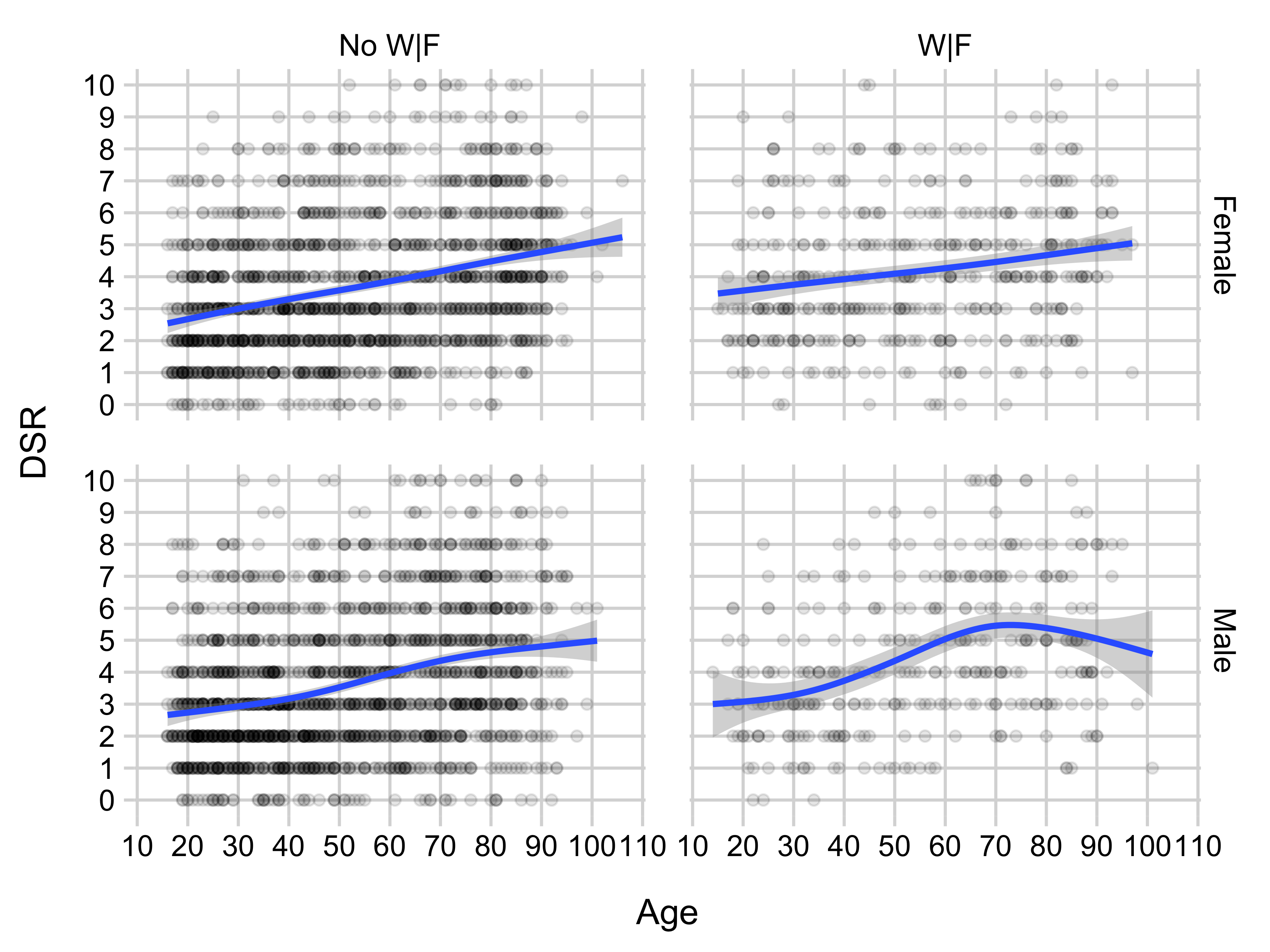

Supplement: S1 Fig — DSR increases with older age, but there is clear excess variance not explained by age. The blue lines and the grey bands show smoothed conditional means and their pointwise 95% confidence interval, respectively, based on generalized additive models. (TIFF) [file pone.0239902.s002.tiff]

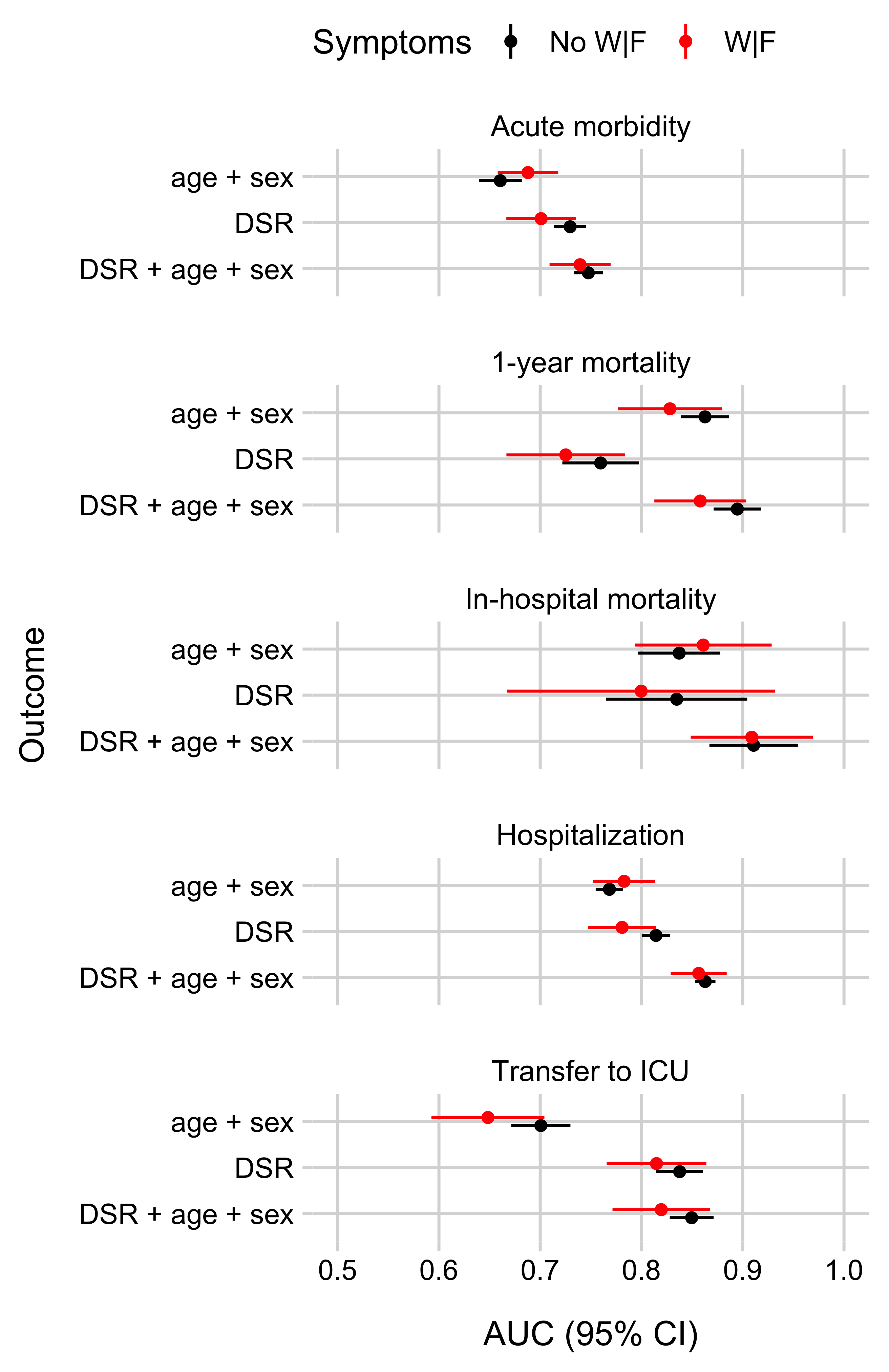

Supplement: S2 Fig — Panel rows show results for the five outcomes: Acute morbidity, 1-year mortality, in-hospital mortality, hospitalization, and transfer to ICU. Each panel shows cross-validated AUC values (plus a 95% confidence interval, CI) for DSR, age, and sex, separately for patients with W|F (in red) and for patients with other symptoms (“No W|F” in black). See Methods for details on how AUC and CI were calculated. (TIFF) [file pone.0239902.s003.tiff]

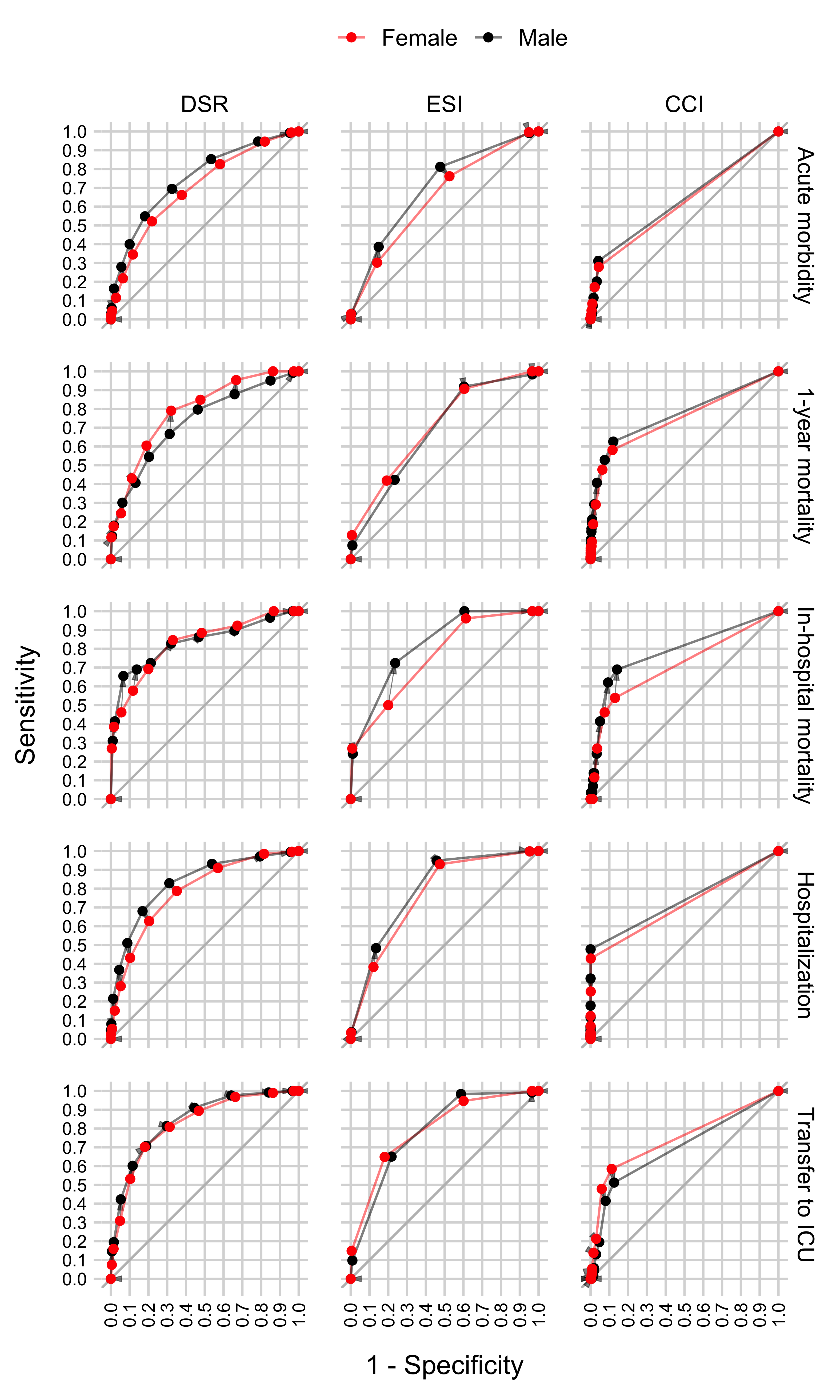

Supplement: S3 Fig — Panel columns show results for the three scores: Disease Severity Rating (DSR), Emergency Severity Index (ESI), and Charlson Comorbidity Index (CCI). Panel rows show results for the five outcomes: Acute morbidity, 1-year mortality, in-hospital mortality, hospitalization, and transfer to ICU. Each panel shows two ROC curves for a particular combination of score and outcome: One ROC curve for female patients (in red) and one ROC curve for male patients (in black). Each point shows the sensitivity (y-axis) and 1 –specificity (x-axis) for each possible thresholding value for a score (i.e., possible ROC operating points). The corresponding operating points for female and male patients are connected by an arrow, which highlights how, if at all, the sensitivity and specificity of the same thresholding value for a particular score differs between the two patient groups. (TIFF) [file pone.0239902.s004.tiff]

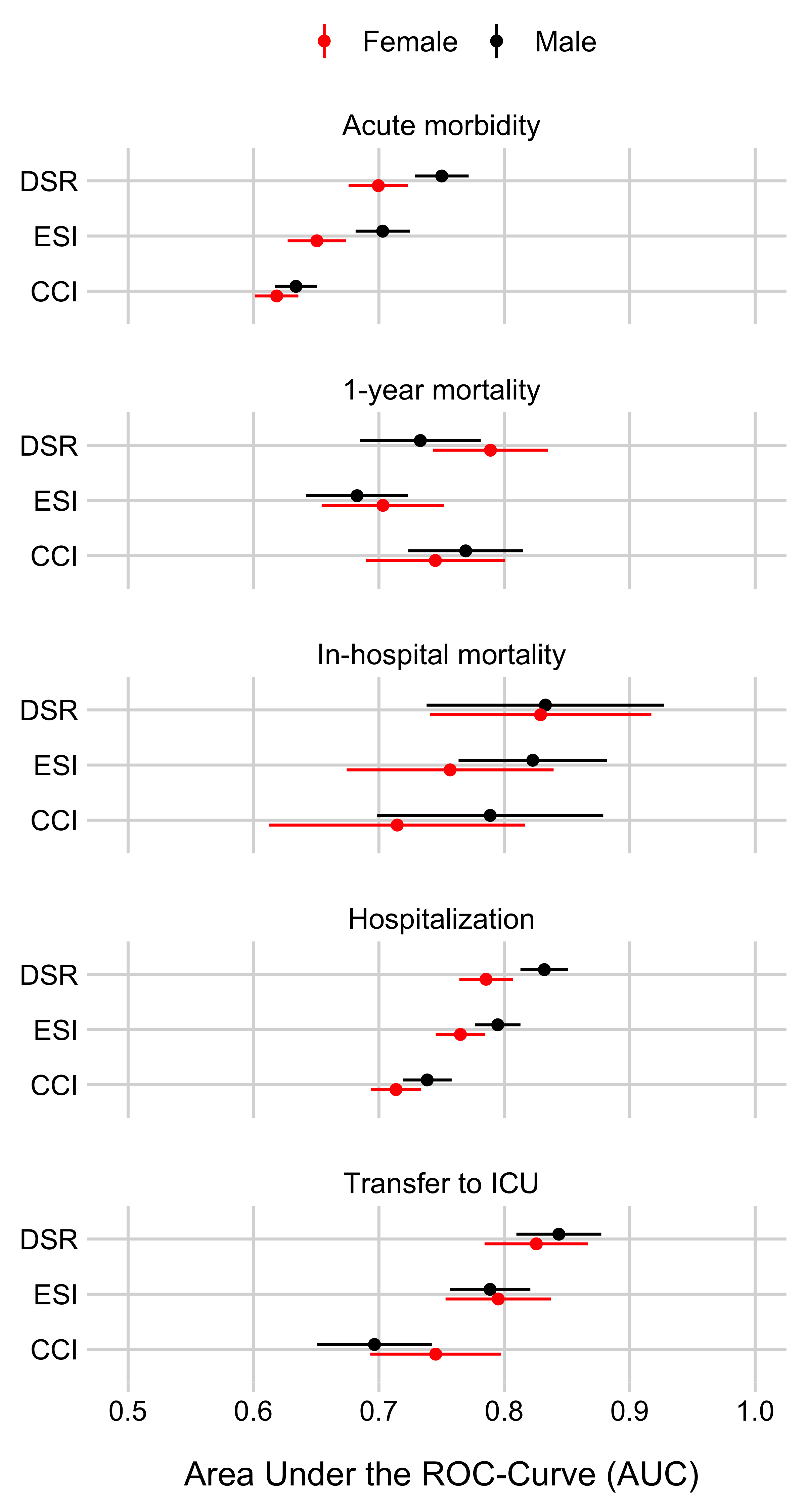

Supplement: S4 Fig — Panel rows show results for the five outcomes: Acute morbidity, 1-year mortality, in-hospital mortality, hospitalization, and transfer to ICU. Each panel shows the in-sample AUC values (plus a 95% confidence interval, CI) for the three scores, Disease Severity Rating (DSR), Emergency Severity Index (ESI), and Charlson Comorbidity Index (CCI), separately for female patients (in red) and male patients (in black). See Methods for details on how AUC and CI were calculated. (TIFF) [file pone.0239902.s005.tiff]
